# Variable precipitation leads to dynamic range limits of forest songbirds at a forest‐grassland ecotone

**DOI:** 10.1002/ece3.7899

**Published:** 2021-07-29

**Authors:** Emily A. Sinnott, Monica Papeş, Timothy J. O’Connell

**Affiliations:** ^1^ School of Natural Resources University of Missouri Columbia Missouri USA; ^2^ Department of Ecology and Evolutionary Biology University of Tennessee Knoxville Tennessee USA; ^3^ Department of Natural Resource Ecology and Management Oklahoma State University Stillwater Oklahoma USA

**Keywords:** citizen science, climate change, historical drought, longitudinal shift, niche modeling, potential distribution

## Abstract

Boundaries between vegetation types, known as ecotones, can be dynamic in response to climatic changes. The North American Great Plains includes a forest‐grassland ecotone in the southcentral United States that has expanded and contracted in recent decades in response to historical periods of drought and pluvial conditions. This dynamic region also marks a western distributional limit for many passerine birds that typically breed in forests of the eastern United States. To better understand the influence that variability can exert on broad‐scale biodiversity, we explored historical longitudinal shifts in the western extent of breeding ranges of eastern forest songbirds in response to the variable climate of the southern Great Plains. We used climatic niche modeling to estimate current distributional limits of nine species of forest‐breeding passerines from 30‐year average climate conditions from 1980 to 2010. During this time, the southern Great Plains experienced an unprecedented wet period without periodic multi‐year droughts that characterized the region's long‐term climate from the early 1900s. Species’ climatic niche models were then projected onto two historical drought periods: 1952–1958 and 1966–1972. Threshold models for each of the three time periods revealed dramatic breeding range contraction and expansion along the forest‐grassland ecotone. Precipitation was the most important climate variable defining breeding ranges of these nine eastern forest songbirds. Range limits extended farther west into southern Great Plains during the more recent pluvial conditions of 1980–2010 and contracted during historical drought periods. An independent dataset from BBS was used to validate 1966–1972 range limit projections. Periods of lower precipitation in the forest‐grassland ecotone are likely responsible for limiting the western extent of eastern forest songbird breeding distributions. Projected increases in temperature and drought conditions in the southern Great Plains associated with climate change may reverse range expansions observed in the past 30 years.

## Graphical abstract

We evaluated distributional shifts of nine eastern forest songbirds at the western edge of their range extent along the transitional ecotone between eastern temperate forests and the central great plains of southcentral United States. Niche modeling of community science data revealed distributional limits of these species are sensitive to variable precipitation and periodic drought events.

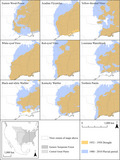

## 1 INTRODUCTION

Zones of changing temperature and precipitation can mark the boundaries of species’ distributions (e.g., Zuckerberg et al., 2009). Studying dynamics at species’ distributional limits is important to understand threshold responses in areas of increased environmental stress (Holt & Keitt, 2005). Individuals at their distributional limits may be especially vulnerable to climate change because they occupy sites with prevailing conditions generally outside their optimal climatic niche (Glennon, 2014; Thuiller et al., 2005). Bird distributions are spatially dynamic in response to changes in temperature and precipitation over time, and this has been seen as evidence of dispersal to and colonization of new areas that fall within a species’ climatic niche (Araujo et al., 2005; Huang et al., 2017; Tingley et al., 2009).

The influence of temperature on bird distributions has been well studied. In North America and Europe, some bird species and assemblages have shifted their latitudinal range limits and mean distributions northward in response to warming temperatures (Devictor et al., 2008; Hitch & Leberg, 2006; Thomas & Lennon, 1999; Zuckerberg et al., 2009). However, not all species exhibit significant distributional shifts in response to temperature, and some that do exhibit multidirectional shifts in response to precipitation or other environmental influencers (Currie & Venne, 2017; Huang et al., 2017). For example, breeding distribution of Henslow's Sparrow (*Ammodramus henslowii*) in the temperate United States has not shifted northward in response to rising temperature, suggesting other factors, including changes in precipitation, might be better predictors of range shifts for grassland birds (McCauley et al., 2017). Drought‐sensitive grassland birds in the southern Great Plains vary in their responses according to the temporal scale of droughts (Cady et al., 2019). Long‐term data suggest some bird species are responsive to local climate changes and may shift their distributions in response to temperature, precipitation, or both depending on which climatic variable limits Net Primary Productivity (Tingley et al., 2009). Thus, there is potential for multiple climatic variables, including temperature, precipitation, and potential evapotranspiration (PET), to exert a strong influence over species’ distributions (Barbet‐Massin & Jetz, 2014).

**FIGURE 1:**
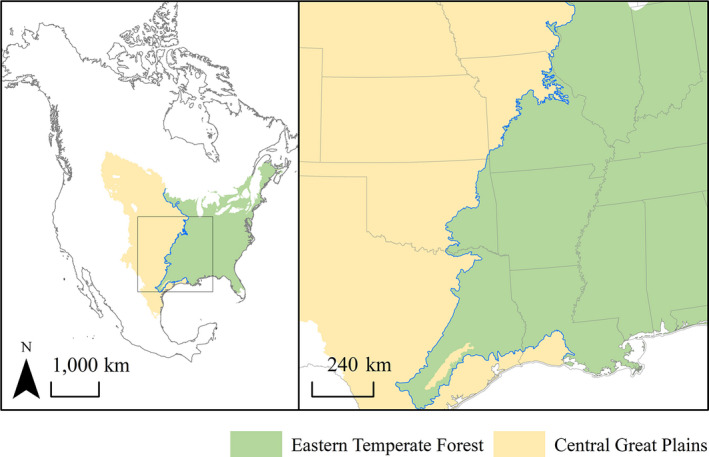
Map of the study area illustrating the transitional ecoregion between eastern oak‐hickory forests and the grasslands of the Great Plains

**FIGURE 2:**
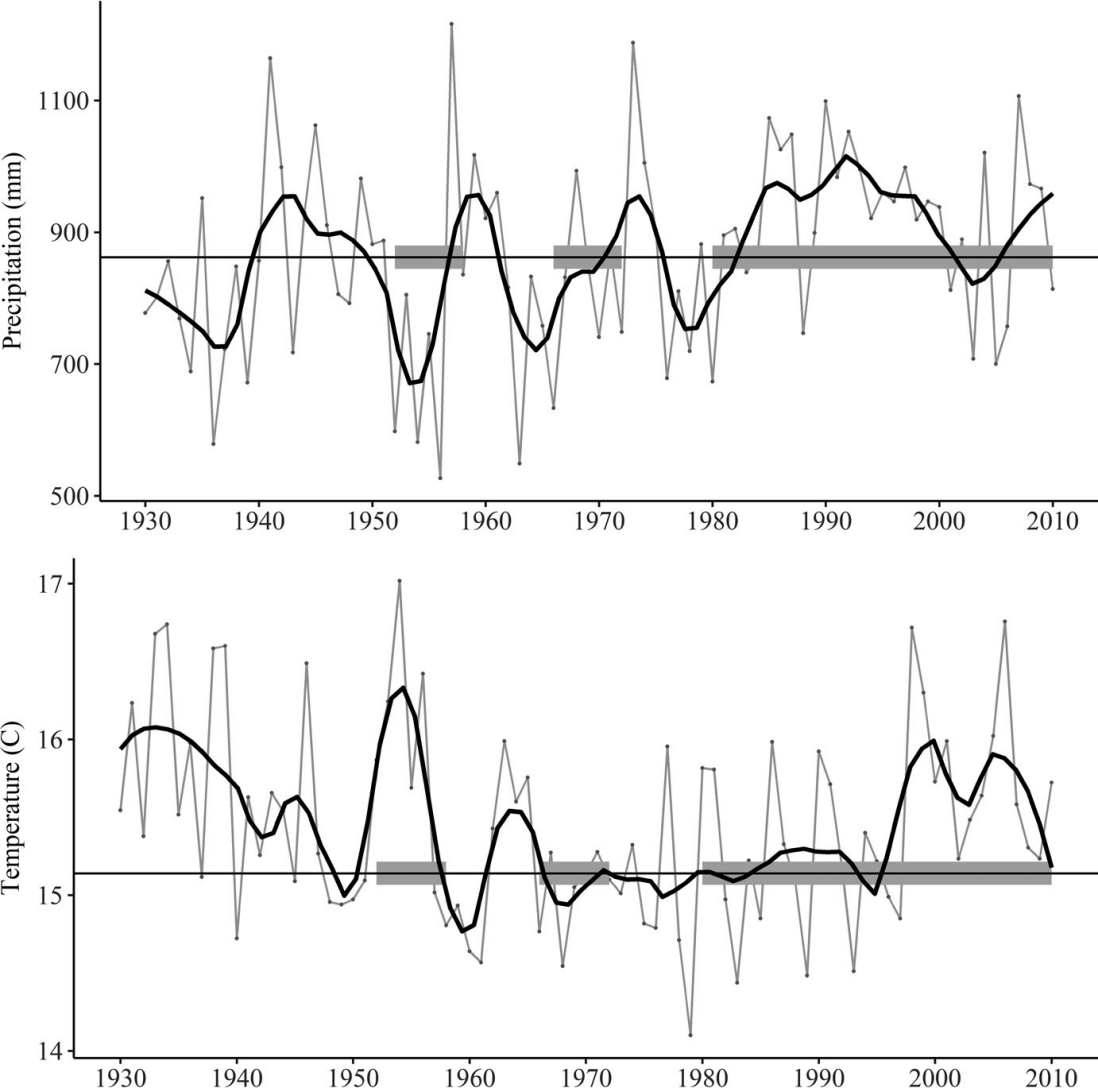
Annual precipitation and temperature trends for the state of Oklahoma 1930–2015. The fine gray points symbolize annual climate values, while the black trend lines are 5‐year moving averages of annual climate conditions. The horizontal line represents mean precipitation and temperature conditions 1930–2015, and the three study periods are highlighted by a thick gray line. Major multi‐year droughts occurred in 1930–1939 and 1952–1958, and an extended dry period occurred in 1962–1972 (LSU 2012)

In the southcentral United States, a marked precipitation gradient defines the broad ecotone between temperate forests of eastern North America and grasslands of the central Great Plains. A transitional landscape characterizes this region between the oak‐hickory (*Quercus* spp. and *Carya* spp.) forests and the tallgrass prairies of the eastern Great Plains (Figure 1). Subject to periodic droughts and traditionally managed with fire and grazing, this region blends oak woodland, oak savanna, and tallgrass prairie vegetation (Rice & Penfound, 1950; Fuhlendorf et al., 2009). Long‐term precipitation trends in the Great Plains are highly variable, and severe extended droughts have alternated with pluvial periods (Figure 2; Basara et al., 2013). Fire suppression has also changed forest composition and structure since the 1950s; stand density has increased, and mesophytic species such as elms (*Ulmus spp*.), red mulberry (*Morus rubra*), and eastern redcedar (*Juniperus virginiana*) have expanded farther west (DeSantis et al., 2010). Decades of fire suppression and periods of above‐average precipitation have contributed to the recent widespread conversion of oak savanna to closed‐canopy forest patches (DeSantis et al., 2010). The dynamic transitional ecoregion of the southcentral United States is well suited for examining the role of changing climate on the distributions of native organisms.

Many eastern forest songbirds reach the western extent of their breeding range in these transitional forests (Heinen & O’Connell, 2009; Reinking, 2004). These species may be sensitive to climate fluctuations at the forest‐grassland ecotone (Clement et al., 2019). Based on Breeding Bird Survey population trends since 1966, the abundance of at least 15 eastern forest birds has increased and expanded westward into the southern Great Plains (Sauer et al., 2014). Expansion of these forest songbirds may have been facilitated by the 1980–2010 pluvial climate and the expansion of mesophytic tree species.

In the southern Great Plains, extended severe droughts occurred in the 1930s and 1950s. In contrast, between 1980 and 2010 an unusually wet climate prevailed in Oklahoma (Figure 2; Basara et al., 2013). The southern Great Plains are projected to have more frequent and extended drought events in the near future (Shafer et al., 2014). To predict species’ responses to projected climate change, it is important to understand historical responses to fluctuating and extreme climatic conditions (Araujo & Pearson, 2005). Our objectives were to (1) examine how western distributional limits of eastern forest songbirds shifted from historical drought periods to recent pluvial conditions and to (2) verify the reliability of historical projections of potential species distributions. We trained a climatic niche model for nine eastern forest songbirds using presence‐only data from eBird and BBS from 1980 to 2010 and concurrent average climate data, and then projected the model onto 1952–1958 and 1966–1972 climate rasters, the latter evaluated with North American Breeding Bird Survey (BBS) data from 1966 to 1972. We hypothesized that historical droughts were responsible for longitudinal retractions of breeding distributions eastward and that pluvial periods created opportunities for expansion westward into the Great Plains.

## 2 METHODS

### 2.1 Bird species records

We generated potential distribution maps of nine passerine species that nest broadly across forested landscapes of eastern North America, reaching a western distributional limit in the Great Plains. We used presence‐only data from the community science network, eBird, and from the North American Breeding Bird Survey (BBS; Cooper et al., 2014; Sullivan et al., 2009). eBird is a community science project established in 2002 that engages millions of avocational bird watchers in the collection of semistructured data on the distribution and abundance of birds (Sullivan et al., 2014). The BBS is a standardized and systematic survey using specific methods and highly trained observers to estimate the abundance and trends of breeding birds in the United States, southern Canada, and northern Mexico. The BBS is a roadside survey established in 1966. BBS routes include 50 stops approximately 0.8 km apart where trained observers record counts of species detected during a 3‐min listening period (Sauer et al., 2014). Each BBS route includes observation aggregations at five ten‐stop segments.

Presence data from eBird and the BBS were downloaded for Eastern Wood‐Pewee (*Contopus virens*), Acadian Flycatcher (*Empidonax virescens*), White‐eyed Vireo (*Vireo griseus*), Yellow‐throated Vireo (*Vireo flavifrons*), Red‐eyed Vireo (*Vireo olivaceus*), Louisiana Waterthrush (*Parkesia motacilla*), Black‐and‐white Warbler (*Mniotilta varia*), Kentucky Warbler (*Geothlypis formosa*), and Northern Parula (*Setophaga americana*). We selected presence data between 1980 and 2010 within the eastern temperate forest and central Great Plains ecoregions of the United States to match the extent and time frame of climate data used for training our models of species’ climatic niches. From the BBS, we obtained presence data from routes in which species were detected within the first 10 stops (8 km) of each route. Locations associated with BBS presence data were assigned to the starting point of each survey route. To match the effort and resolution of eBird presence data with BBS 10 stop data, we selected eBird presence data between June and July from checklists that covered less than 8 km in less than 60 min with fewer than 10 observers (Strimas‐Mackey et al., 2020). To reduce sampling bias in species climatic niche models, we spatially rarefied occurrence data by 40 km using SDMtoolbox (Boria et al., 2014; Brown, 2014).

### 2.2 Climate variables

We retrieved climatic data from the PRISM Climate Group models (PRISM Climate Group, 2014). Environmental variables included annual cumulative precipitation, annual mean temperature, and spring (April–June) and summer (July–August) PET at 4km resolution (PRISM Climate Group, 2014; Thornthwaite, 1948). These environmental variables were selected as consistently relevant across bird species when developing species distribution models (Barbet‐Massin & Jetz, 2014). Temperature, precipitation, and PET values were standardized based on their mean and standard deviation. We used average climate data across years for each of three time periods: 1980–2010, 1966–1972, and 1952–1958 using ArcGIS 10.7.1 (ESRI, 2011, Redlands, California, USA). All climate variables were clipped to the extent of the eastern temperate forest and central Great Plains ecoregions of the United States to train and project species’ climatic niche models.

### 2.3 Maxent modeling parameters and evaluation

We modeled species’ climatic niches in the program Maxent 3.4.4 using presence‐only occurrences and climate data (Phillips et al., 2006; Phillips & Dudick, 2008). The Maxent algorithm estimates environmental parameters of a species’ niche by sampling occurrences from its known distribution and contrasting them with randomly selected background samples (Elith et al., 2006; Phillips et al., 2006). For our models, we used the default Maxent ver. 3.4.4 settings for background sampling (10,000), as well as for features (environmental constraints of probability distribution), regularization multiplier, and maximum iterations and convergence threshold. We randomly selected 70% of species’ occurrences to train models and generate potential distribution maps from the 1980–2010 average climate data and 30% of occurrence points to test model performance on the 1980–2010 distribution map. A first set of models was run using all variables; variables that contributed less than 1.0% to overall accuracy gain in the initial model were removed from the final model. We evaluated the accuracy of 1980–2010 models by calculating the continuous Boyce index on the random 30% of presence data withheld as test points (Hirzel et al., 2006). Boyce index was calculated in program R version 4.1.0 using the package ecospat (Broennimann et al., 2021; R Core Team, 2021). The continuous Boyce index ranges from −1 to 1. Positive Boyce index values indicate that model predictions are consistent with species presences from test data, while values close to zero or negative indicate that models predict poorly species presence based on test data (Hirzel et al., 2006). Omission error (percentage of test presences predicted absent) was also calculated to evaluate model performance. To calculate omission error, we converted Maxent outputs of probability suitability values between 0 and 1 to binary (suitable–unsuitable) maps by applying the 10‐percentile training presence threshold rule. This rule selects the Maxent value at which 10% of the occurrence locations used to train the model are predicted climatically unsuitable and then reclassifies all locations with values below this threshold as unsuitable and those above the threshold as suitable. The models were projected onto the 1952–1958 and 1966–1972 climates, and we applied the same 10% threshold rule to generate binary suitable–unsuitable potential distribution maps for each time period. We used these maps to identify shifts in species range boundaries.

### 2.4 Independent validation of historical projections

We used BBS presence data from the first 10 stops of 1966–1972 roadside surveys and locations of route starting points as independent evaluation points to indicate the predictive power of historical projections of species’ potential climatic distributions (Pardieck et al., 2015). We evaluated the reliability of 1966–1972 species’ estimated potential distributions by calculating the continuous Boyce index on concurrent presence data from BBS. This validation was not possible for model projections of 1952–1958 potential distributions due to the lack of independent datasets of species’ presences for that time frame; thus, the model performance is the only indicator of reliability of these estimates.

## 3 RESULTS

**FIGURE 3:**
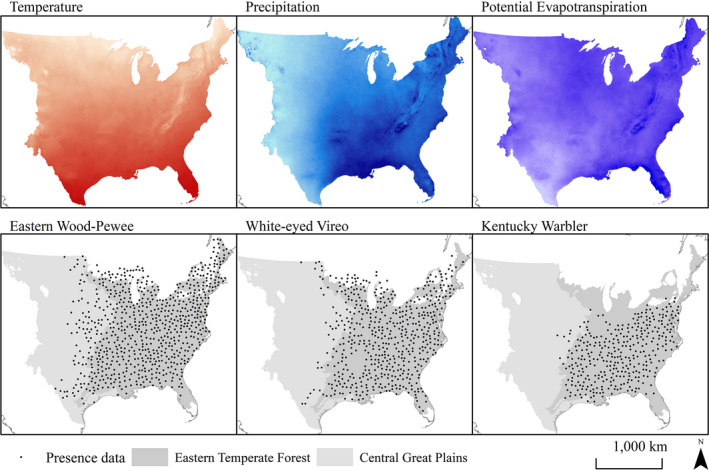
Climate and species presence data used to generate climatic niche models for nine eastern forest songbirds, including Eastern Wood‐Pewee, White‐eyed Vireo, and Kentucky Warbler. Climate variables included average annual temperature and precipitation and spring and summer potential evapotranspiration retrieved from PRISM climate group. Presence data were retrieved from eBird and Breeding Bird Survey records from June and July 1980–2010 and then spatially rarefied at 40km

After spatially filtering eBird and BBS presence data to 40 km, species’ climatic niche models were generated from 740 occurrences for Eastern Wood‐Pewee, 453 for Acadian Flycatcher, 557 for White‐eyed Vireo, 547 for Yellow‐throated Vireo, 794 for Red‐eyed Vireo, 297 for Louisiana Waterthrush, 400 for Black‐and‐white Warbler, 348 for Kentucky Warbler, and 474 occurrences for Northern Parula (Figure 3). Continuous Boyce index values for the 1980–2010 test data ranged from 0.546 for Eastern Wood‐Pewee to 0.981 for White‐eyed Vireo (Table 1), while omission error ranged from 0.108 for Black‐and‐white Warbler to 0.157 for Louisiana Waterthrush, thus both metrics indicating good model performance. Based on Maxent‐derived percent contribution of climate variables to model accuracy gain, annual precipitation was the most important contributor to all species’ climatic niche models except for White‐eyed Vireo, for which average annual temperature had the greatest contribution to the Maxent model (Table 2). Percent contribution of precipitation was greatest for Northern Parula (94.7%) and Eastern Wood‐Pewee (89.3%), while percent contribution of temperature variables was greatest for White‐eyed Vireo (52.4%) and Kentucky Warbler (23.2%). Potential evapotranspiration had a limited contribution to climatic niche models and was greatest for Yellow‐throated Vireo (11.3%) and Black‐and‐white Warbler (9.6%; Table 2).

**TABLE 1:** Sample size of presence data and performance metrics of climatic niche models estimating potential distributions of nine eastern forest songbirds

	1980–2010				1966–1972		
	*N*_Train_	*N*_Test_	Boyce Index	Omission	*N*_Test_	Boyce Index	Omission
Eastern Wood‐Pewee	518	222	0.546	0.135	760	0.981	0.076
Acadian Flycatcher	318	135	0.941	0.148	218	0.976	0.073
White‐eyed Vireo	390	167	0.981	0.144	376	0.870	0.056
Yellow‐throated Vireo	383	164	0.933	0.146	260	0.838	0.046
Red‐eyed Vireo	556	238	0.862	0.147	743	0.979	0.057
Louisiana Waterthrush	208	89	0.941	0.157	98	0.902	0.122
Black‐and‐white Warbler	280	120	0.964	0.108	237	0.928	0.034
Kentucky Warbler	244	104	0.921	0.115	184	0.748	0.130
Northern Parula	332	142	0.825	0.141	153	0.943	0.092

Notes

The number of observations used to train each climate niche model on 1980–2010 climate conditions (N_Train_) is given as well as the number of testing observations used as evaluation points to calculate the Boyce index and Omission rates for 1980–2010 and 1966–1972 predictions (N_Test_). Continuous Boyce index was calculated as a metric of model accuracy; values close to 1 indicate consistency between independent presence data and estimated species distributions, whereas values close to zero or negative indicate poor predictive capacity. Omission rate, another metric of model accuracy, was calculated from the 10% training presence threshold models.

**TABLE 2:** Percent contribution of climate variables to accuracy gain of species climatic niche models for nine eastern forest songbirds

	P	T	PET_spring_	PET_summer_
Eastern Wood‐Pewee	0.893	0.079	0.027	‐
Acadian Flycatcher	0.829	0.171	.	0.017
White‐eyed Vireo	0.459	0.524	.	
Yellow‐throated Vireo	0.854	0.034	.	0.113
Red‐eyed Vireo	0.86	0.057	.	0.084
Louisiana Waterthrush	0.82	0.145	.	0.035
Black‐and‐white Warbler	0.734	0.17	0.096	.
Kentucky Warbler	0.745	0.232	0.023	.
Northern Parula	0.947	0.034	.	0.019

Notes

Presence‐only data from 1980–2010 were related to concurrent average annual precipitation (P) and temperature (T), as well as spring and summer potential evapotranspiration (PET) across the eastern temperate forest and central Great Plains ecoregions of the United States.

“.” Indicates a variable contributed <1% to a climatic niche model in the full model and was not included in the final reduced model.

**FIGURE 4:**
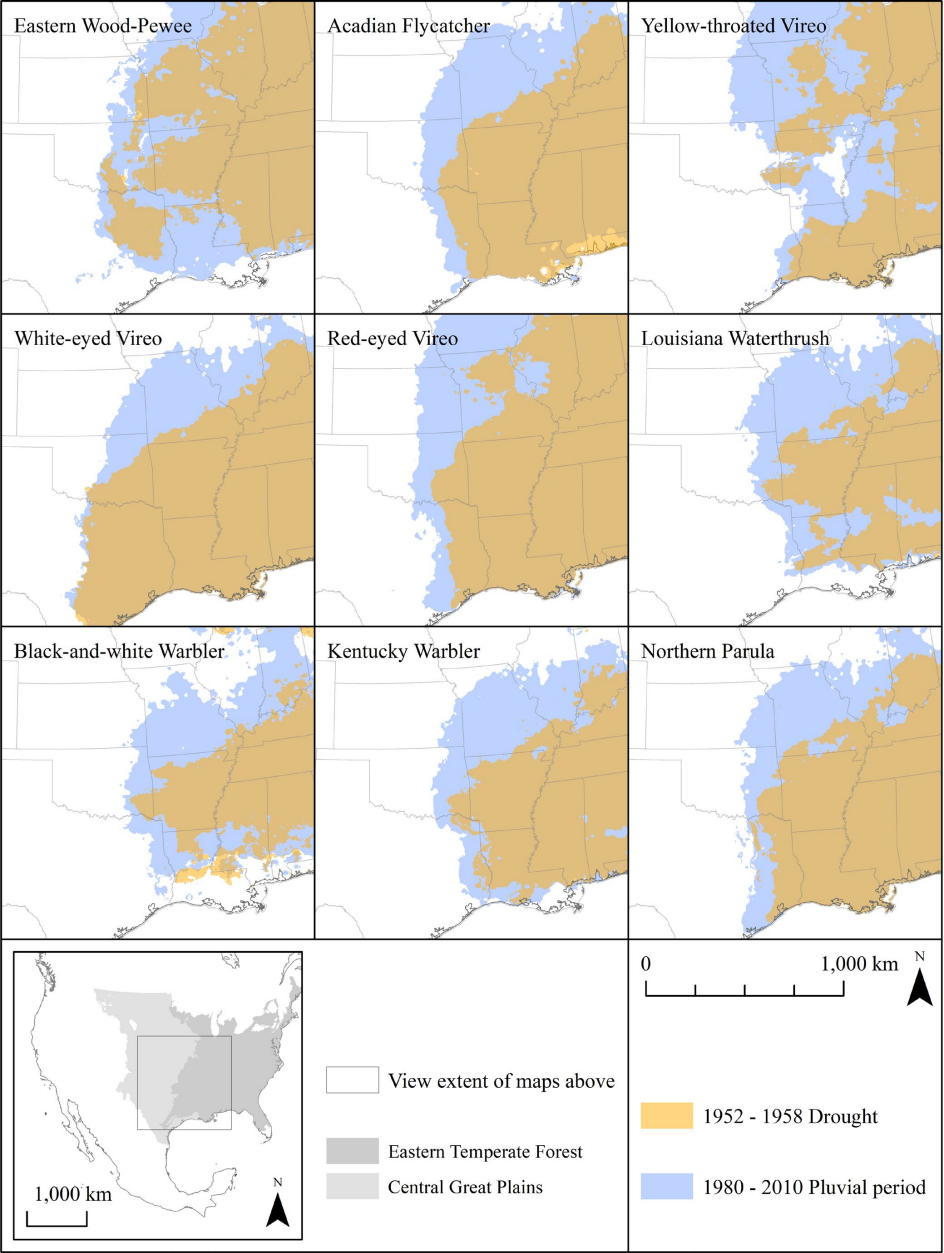
Potential species distributions estimated with Maxent models, showing westward shifts in species estimated range margins from the severe drought of 1952‒1958 (red) to the pluvial period of 1980‒2010 (blue) for Eastern Wood‐Pewee, Acadian Flycatcher, White‐eyed Vireo, Yellow‐throated Vireo, Red‐eyed Vireo, Louisiana Waterthrush, Black‐and‐white Warbler, Kentucky Warbler, and Northern Parula

**FIGURE 5:**
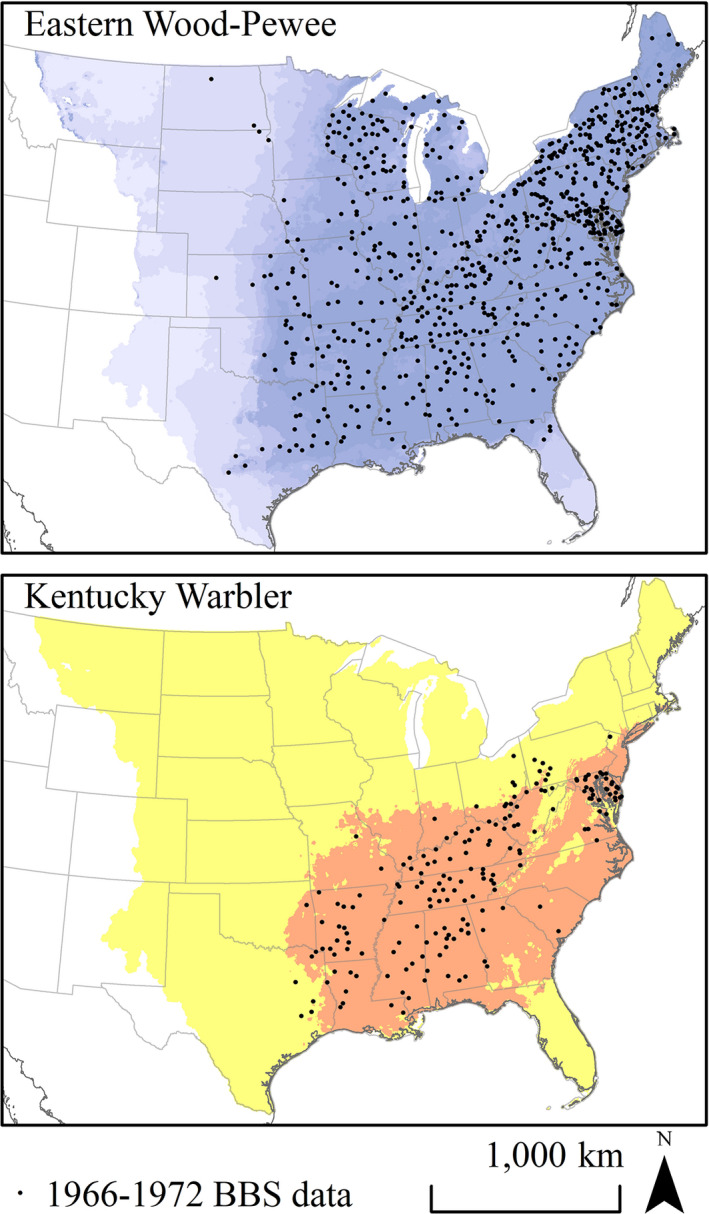
Maps illustrating climatic niche models trained using eBird and Breeding Bird Survey (BBS) data from 1980‒2010 and climate data for the same timeframe estimated reliably historical distributions of species from 1966‒1972. Top: Probability of suitability of Eastern Wood‐Pewee 1966‒1972 across the eastern temperate forests and central Great Plains based on historical climate projections of climatic niche model, overlaid with independent presence data from Breeding Bird Survey (BBS) routes surveyed 1966‒1972. Bottom: Binary map illustrating predicted distributional limits of Kentucky Warbler from 1966‒1972 estimated using 10% threshold of training presence data overlaid with independent presence data from BBS routes surveyed 1966‒1972

The southwestern limit of all species’ potential distributions during 1952–1958 historical drought years contracted farther east from recent pluvial conditions (Figure 4). Severe historical droughts resulted in the longitudinal retraction of species’ estimated western breeding distributions in the southern Great Plains. We assessed the reliability of species’ estimated historical distributions during drought years using independent data from the 1966–1972 BBS surveys as test data on projected potential distributions. Continuous Boyce index values for historical estimated potential distributions ranged from 0.748 for Kentucky Warbler to 0.981 for Eastern Wood‐Pewee, suggesting reasonably reliable estimates (Table 1). Omission error for historical estimated potential distributions ranged from 0.034 for Black‐and‐White Warbler and 0.130 for Kentucky Warbler (Table 1, Figure 5).

## 4 DISCUSSION

We found that changes in climate conditions resulted in longitudinal shifts in estimated potential distributions of eastern forest songbirds along a broad forest‐grassland ecotone. Our results describe westward expansion of species’ potential distributions from historical drought conditions in 1952–1958 and 1966–1972, to recent pluvial conditions in 1980–2010 in the southcentral United States. These results are consistent with at least one study that found high rates of colonization and a positive occupancy trend for Eastern Wood‐Pewee in the southwest portion of its range in the late 1990s (Clement et al., 2019). Additionally, White‐eyed Vireo, Red‐eyed Vireo, and Louisiana Waterthrush have increased in abundance through much of their range in the southcentral United States since 1966, while Black‐and‐white Warbler, Kentucky Warbler, and Northern Parula have increased locally in that period (Sauer et al., 2014).

We found the western limits of potential distributions of eastern forest songbirds along a forest‐grassland ecotone were sensitive to precipitation and temperature changes under historical drought conditions. Precipitation most strongly influenced the potential distributions for Eastern Wood‐Pewee, Acadian Flycatcher, Yellow‐throated Vireo, Red‐eyed Vireo, Louisiana Waterthrush, Black‐and‐white Warbler, Kentucky Warbler, and Northern Parula. Temperature most strongly influenced the potential distribution for White‐eyed Vireo and was an important contributor to other species climate niche models, including that of Kentucky Warbler. Our models agree with previous efforts to link local climatic conditions to patterns of colonization and extinction that underlie changes in species distributions. For example, dynamic occupancy models suggest that colonization of new areas by Louisiana Waterthrush was associated with moderate precipitation and extinction was associated with extreme temperatures (Clement et al., 2016). Additionally, changes in total annual periods of extreme temperatures influenced colonization and extinction rates for both Eastern Wood‐Pewee and Red‐eyed Vireo (Clement et al., 2019).

Specific mechanisms of potential expansion and contraction of breeding distributions were beyond the scope of this study, but potentially associated with changes in vegetation structure and composition. Ecotone shifts are driven by climate patterns (Allen & Breshears, 1998; DeSantis et al., 2010; Risser, 1995). Changes in vegetation affected by variable precipitation can alter bird community composition. Since the 1950s, fire suppression and a wetter climate are correlated with the mesophication of transitional savannas between the southern Great Plains and the eastern temperate forests (DeSantis et al., 2010). Riparian gallery forests have expanded into tallgrass prairies of the southern Great Plans (Wine & Zhou, 2012). Tree species richness, as well as the basal area and tree density of eastern red cedar, elms, red mulberry, black hickory, and sugarberry have increased (DeSantis et al., 2010). During this period of mesophication, many eastern forest songbirds expanded farther west into the southern Great Plains (Sauer et al., 2014).

Structural attributes of forests are important determinants of forest bird density (Bakermans et al., 2012). Forest cover, soil moisture, and canopy height are among the most important environmental predictors shaping songbird community composition in this transitional ecotone (Cavalieri et al., 2009). Increased precipitation during pluvial periods may have contributed to changes in forest composition, structure, and extent at the forest‐grassland ecotone, thereby facilitating the recent western expansion of eastern forest songbirds’ breeding range. Among the focal species included in this study, Kentucky Warbler is positively associated with increased ground litter, understory density, and canopy height (Bakermans et al., 2012). Eastern Wood‐Pewee occupies forests with greater tree density and canopy cover and landscapes with greater forest cover overall (Holoubek & Jensen, 2015; Kendrick et al., 2013). Acadian Flycatcher, Northern Parula, and Red‐eyed Vireo are mature forest species associated with higher canopies (Reidy et al., 2014). Forest patches, especially expanding from riparian gallery forests, have increased the amount of land area in the southern Great Plains with attributes that are potentially attractive to bird species typically associated with temperate forests of eastern North America (Knight et al., 1994).

Although pluvial periods can create conditions amenable to the expansion of forest birds in the Great Plains, droughts can have the opposite effect. Water‐limited forests are especially vulnerable to temperature increases and drought which can increase the background rate of tree mortality, cause widespread forest die‐off, and induce rapid shifts in woodland ecotones (Allen & Breshears, 1998; Allen et al., 2010; Breshears et al., 2005; van Mantgem et al., 2009). Drought events have been responsible for oak mortality in savannas of Minnesota and oak decline in the Ozarks of Missouri (Faber‐Langendoen & Tester, 1993; Voelker et al., 2008). In the 1950s, drought stress caused significant tree mortality in the oak savannas of central Oklahoma, where 11.8% of trees died within a stand (Rice & Penfound, 1959). The forest‐grassland boundary of the southcentral United States is also sensitive to fluctuating trends in precipitation and temperature, where extended droughts have caused high rates of tree mortality and canopy loss in transitional oak woodlands and forests (Albertson & Weaver, 1945; Rice & Penfound, 1959; Schwantes et al., 2017).

Historical trends in climate and concurrent shifts in the range margins of eastern forest songbirds provide insights into species’ distributional responses to projected climate changes in the Great Plains. Across the Great Plains, 2011 had the third highest number of heat waves in recorded history, just behind the extreme drought years of 1934 and 1936 (Kunkel et al., 2013). Climate simulations project an increase in the number of days with temperatures over 35˚C, an increase in average annual temperature, and an increase in summer temperatures in the southern Great Plains (Kunkel et al., 2014; NARCCAP, 2012). The southern Great Plains is projected to become drier as well. In Oklahoma, summer precipitation is projected to decrease and the number of dry days is projected to increase (Kunkel et al., 2014).

Cause and effect relationships are difficult to determine and both climate and land cover change drive distributional changes for many bird species (Clement et al., 2019). We examined changes in potential distributions of eastern forest songbirds based on projections of climatic niche models onto historical climate conditions. We did not evaluate contributions of land cover change to species distributional shifts. We also could not disentangle the confounding effects of increased precipitation and fire suppression on forest cover and structure (Wine & Zhou, 2012). Lastly, not all species track changes in temperature over time across their geographic distributions and species’ realized temperature niche may change over time (Currie & Venne, 2017).

Our projections of eastern forest species’ distributions to historical drought conditions suggest that observed range expansion in portions of the southern Great Plains could reverse under the warmer and drier conditions forecasted for this region. Our comparison of model‐based estimates of historical distributions and the early BBS data indicated that climatic variables were fairly reliable at estimating distributional extents of eastern forest species during the dry conditions of 1966–1972. This adds confidence to the range margin shifts projected in our historical 1950s drought scenario. Although the temperature is often seen as the primary driver of climatic change effects on wildlife, in our study the low predictive power of this driver in regional and individual species’ responses highlights the need for more case‐by‐case research than for broad generalities (Currie & Venne, 2017). For forest‐breeding passerines at the edge of their distribution within the transitional ecotone between the southern Great Plains and eastern temperate forests, our results suggest that dynamic trends in precipitation will likely induce greater variability in longitudinal shifts in distribution than temperature increases will affect shifts in latitude. Thus, important information need moving forward will be regional climatic vulnerabilities and the degree to which changes in distributional limits will affect the trajectory of total populations of eastern forest passerines.

## CONFLICT OF INTEREST

The authors declare no conflicts of interest.

## AUTHOR CONTRIBUTION

**Emily Sinnott:** Conceptualization (equal); Data curation (lead); Formal analysis (equal); Investigation (equal); Methodology (equal); Writing‐original draft (lead); Writing‐review & editing (equal). **Timothy O'Connell:** Conceptualization (equal); Funding acquisition (lead); Investigation (equal); Methodology (equal); Project administration (equal); Resources (equal); Software (equal); Supervision (lead); Validation (equal); Visualization (equal); Writing‐review & editing (equal). **Monica Papes:** Conceptualization (supporting); Data curation (supporting); Formal analysis (equal); Investigation (equal); Methodology (lead); Software (equal); Supervision (equal); Validation (equal); Visualization (equal); Writing‐review & editing (equal).

### OPEN RESEARCH BADGES

This article has earned an Open Data, Open Materials Badge for making publicly available the digitally‐shareable data necessary to reproduce the reported results. The data is available at https://doi.org/10.5061/dryad.dfn2z3525; http://biodiversityinformatics.amnh.org/open_source/maxent/, respectively.

## Data Availability

All data used in these climate niche models are publicly available. Occurrence data were requested through eBird and collected from their Basic Dataset (EBD) at http://ebird.org/ebird/data/download. Historical and 30‐year normal climate data were retrieved online through PRISM Climate Group at http://www.prism.oregonstate.edu/.
